# A single-cell map of antisense oligonucleotide activity in the brain

**DOI:** 10.1093/nar/gkad371

**Published:** 2023-05-16

**Authors:** Meredith A Mortberg, Juliana E Gentile, Naeem M Nadaf, Charles Vanderburg, Sean Simmons, Dan Dubinsky, Adam Slamin, Salome Maldonado, Caroline L Petersen, Nichole Jones, Holly B Kordasiewicz, Hien T Zhao, Sonia M Vallabh, Eric Vallabh Minikel

**Affiliations:** Stanley Center for Psychiatric Research, Broad Institute of MIT and Harvard, Cambridge, MA 02142, USA; Stanley Center for Psychiatric Research, Broad Institute of MIT and Harvard, Cambridge, MA 02142, USA; Stanley Center for Psychiatric Research, Broad Institute of MIT and Harvard, Cambridge, MA 02142, USA; Stanley Center for Psychiatric Research, Broad Institute of MIT and Harvard, Cambridge, MA 02142, USA; Stanley Center for Psychiatric Research, Broad Institute of MIT and Harvard, Cambridge, MA 02142, USA; Genomics Platform, Broad Institute of MIT and Harvard, Cambridge, MA 02141, USA; Genomics Platform, Broad Institute of MIT and Harvard, Cambridge, MA 02141, USA; Genomics Platform, Broad Institute of MIT and Harvard, Cambridge, MA 02141, USA; Genomics Platform, Broad Institute of MIT and Harvard, Cambridge, MA 02141, USA; Genomics Platform, Broad Institute of MIT and Harvard, Cambridge, MA 02141, USA; Ionis Pharmaceuticals, Carlsbad, CA 92010, USA; Ionis Pharmaceuticals, Carlsbad, CA 92010, USA; Stanley Center for Psychiatric Research, Broad Institute of MIT and Harvard, Cambridge, MA 02142, USA; McCance Center for Brain Health and Department of Neurology, Massachusetts General Hospital, Boston, MA 02114, USA; Department of Neurology, Harvard Medical School, Boston, MA02115, USA; Prion Alliance, Cambridge, MA 02139, USA; Stanley Center for Psychiatric Research, Broad Institute of MIT and Harvard, Cambridge, MA 02142, USA; McCance Center for Brain Health and Department of Neurology, Massachusetts General Hospital, Boston, MA 02114, USA; Department of Neurology, Harvard Medical School, Boston, MA02115, USA; Prion Alliance, Cambridge, MA 02139, USA

## Abstract

Antisense oligonucleotides (ASOs) dosed into cerebrospinal fluid (CSF) distribute broadly throughout the central nervous system (CNS). By modulating RNA, they hold the promise of targeting root molecular causes of disease and hold potential to treat myriad CNS disorders. Realization of this potential requires that ASOs must be active in the disease-relevant cells, and ideally, that monitorable biomarkers also reflect ASO activity in these cells. The biodistribution and activity of such centrally delivered ASOs have been deeply characterized in rodent and non-human primate (NHP) models, but usually only in bulk tissue, limiting our understanding of the distribution of ASO activity across individual cells and across diverse CNS cell types. Moreover, in human clinical trials, target engagement is usually monitorable only in a single compartment, CSF. We sought a deeper understanding of how individual cells and cell types contribute to bulk tissue signal in the CNS, and how these are linked to CSF biomarker outcomes. We employed single nucleus transcriptomics on tissue from mice treated with RNase H1 ASOs against *Prnp* and *Malat1* and NHPs treated with an ASO against *PRNP*. Pharmacologic activity was observed in every cell type, though sometimes with substantial differences in magnitude. Single cell RNA count distributions implied target RNA suppression in every single sequenced cell, rather than intense knockdown in only some cells. Duration of action up to 12 weeks post-dose differed across cell types, being shorter in microglia than in neurons. Suppression in neurons was generally similar to, or more robust than, the bulk tissue. In macaques, PrP in CSF was lowered 40% in conjunction with *PRNP* knockdown across all cell types including neurons, arguing that a CSF biomarker readout is likely to reflect ASO pharmacodynamic effect in disease-relevant cells in a neuronal disorder. Our results provide a reference dataset for ASO activity distribution in the CNS and establish single nucleus sequencing as a method for evaluating cell type specificity of oligonucleotide therapeutics and other modalities.

## Graphical Abstract

**Graphical Abstract:**
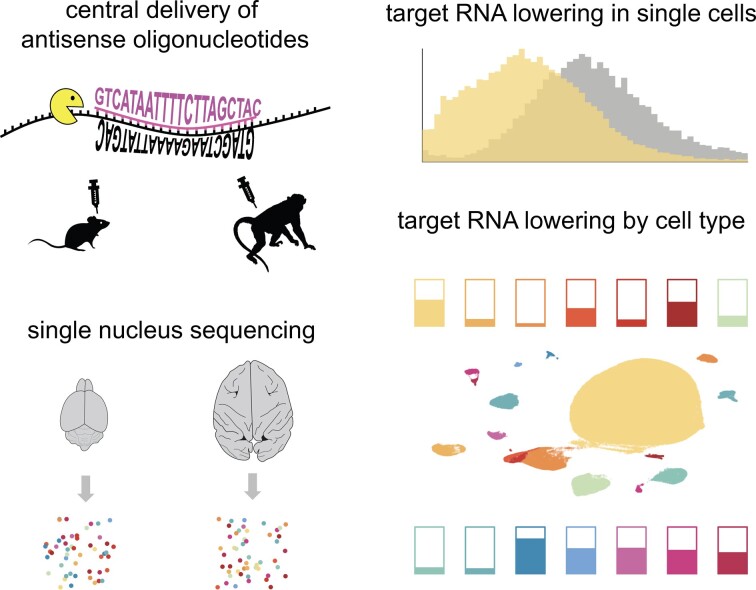

## INTRODUCTION

Antisense oligonucleotides (ASOs) can, in principle, modulate the expression of nearly any gene in the central nervous system (CNS) (1). Bolus injected into cerebrospinal fluid (CSF) (2), ASOs are internalized by cell surface proteins, escape from endosomes (3,4) and become durably active in the cytoplasm and nucleus (5). Since the 2016 approval of nusinersen, an ASO modulator of pre-mRNA splicing in spinal muscular atrophy (6), 23 ASOs have entered trials for CNS disorders (1,7), with several advancing to Phase III, along with others administered on an ‘N-of-1’ basis (8–11). The majority of CNS ASOs in trials today are ‘gapmers’—ASOs with 2' sugar modifications in the wings (typically 5 base pairs on either side) and a ‘gap’ in the middle with no modifications except for a phosphorothioate backbone (2)—designed to lower the expression of a target RNA by recruiting the enzyme RNase H1 to cleave it (12–14). For CNS diseases caused by a toxic gain of function, gapmer ASOs offer a rational approach to target the root cause of disease by lowering the toxic RNA or protein (1).

This rational mechanism is only useful, however, if the drug can be delivered to the right tissues and the right cell types (15). Following the seminal discovery that bolus injection into CSF is an efficient means of delivering ASOs to the CNS (16), several studies have demonstrated drug uptake and durable target RNA silencing for gapmers in rodent and non-human primate (NHP) brain (17–21). Meanwhile, the limited available autopsy data have confirmed that ASOs distribute to multiple regions of spinal cord and brain in humans (11,22). Jafar-nejad and Powers *et al.* in a thorough pharmacology atlas spanning 35 brain regions in NHP, demonstrated a drug concentration gradient, with more accumulation in superficial than in deep brain structures, but at least some target RNA lowering was observed across all regions studied (21). At the bulk tissue level, the above studies demonstrate widespread CNS biodistribution and activity of centrally delivered ASOs.

Available evidence also suggests that ASO activity is broadly distributed across cell types within the CNS. ASOs have been successfully employed to ameliorate CNS disease in animal models with pathologies specific to various cell types including astrocytes, oligodendrocytes, microglia, and several neuronal sub-types (18,23–29). Histological analysis has been used to qualitatively demonstrate ASO uptake and target RNA modulation across diverse CNS cell types in both rodent and NHP (21). Moreover, dose-response relationships were determined for an ASO in four cell types isolated from mouse cortex at two weeks post-dose (21). Thus, whereas rather marked differences in ASO uptake or activity between cell types have been observed in liver and lung (30,31), the above findings suggest relatively broad ASO activity across cell types within the CNS.

Nevertheless, a more comprehensive understanding of how ASO activity is distributed at the cell type and at the single cell level would be a valuable asset. Important knowledge gaps include: how bulk tissue knockdown is distributed across individual cells; quantitative assessment of knockdown across detectable cell types; duration of action by cell type; and ability to translate cell type-specific activity profiles between relevant model systems, such as from mouse to NHP, and from low-potency tool compounds used at high doses versus high-potency compounds used at low doses. These knowledge gaps are particularly salient when considering the interpretation of CSF-based target engagement biomarkers in ASO trials. Cell type-specific differences in ASO uptake or activity, combined with drug concentration gradients, could generate variability in the degree of target engagement among relevant CNS cells, yet biomarker values from a single sampling compartment can underpin choices to advance or halt clinical programs. A deeper profiling of ASO activity across cell types should help to inform such crucial decisions.

We hypothesized that single nucleus RNA sequencing (snRNA-seq) could begin to fill the above knowledge gaps. In particular, transcriptomic information would allow us to assign cell type for each nucleus, providing a relatively unbiased sampling of cells in a tissue. Comparing the number of target RNA counts within those transcriptomes for ASO versus vehicle-treated animals could then be used to quantify target engagement for any group of cells, such as a cell type or subtype, and could even provide information about the distribution of activity across single cells. Here, we employed snRNA-seq to refine our understanding of ASO activity in the mouse and cynomolgus macaque CNS. Our results illuminate the broadness of RNase H1 ASO target engagement across individual cells and across cell types, reveal cell type-specific differences in extent of target RNA lowering and in duration of action, link neuronal target engagement to a CSF biomarker outcome, and establish a reference dataset and a methodology for assessing the cell type specificity of oligonucleotide therapies.

## MATERIALS AND METHODS

### Mice

All mice were female C57BL/6N. Animals for 3 week post-dose harvest were dosed at the Broad Institute (IACUC protocol 0162–05-17) and were 16 weeks old at the time of dosing. Animals for 2 and 12 week post-dose harvest were dosed at Ionis Pharmaceuticals (IACUC protocol 2021–1176) and were 8–12 weeks old at dosing. Mice were dosed via intracerebroventricular injection as described (32). ASOs were delivered as a single bolus injection of 500 μg (*Prnp* ASOs) or 50 μg (*Malat1* ASO) formulated in a 10 μl volume of dPBS. Mice were perfused with HEPES-sucrose solution (110 mM NaCl, 10 mM HEPES, 25 mM glucose, 75 mM sucrose, 7.5 mM MgCl_2_, 2.5 mM KCl, pH 7.4) and brains harvested as described (33,34).

### Non-human primates

Cynomolgus macaque (*Macaca fascicularis*) studies were performed at Labcorp Early Development Services GmbH (Münster, Germany) under IACUC protocol 8422120. Studies complied with all of the following regulations: European Directive 2001/83/EC, German Drug Law Arzneimittelgesetz, International Conference on Harmonization (ICH) gudelines M3(R2) (Guidance on Nonclinical Safety Studies for the Conduct of Human Clinical Trials and Marketing Authorization for Pharmaceuticals), ICH-S3A (Toxicokinetics: A Guidance for Assessing Systemic Exposure in Toxicology Studies), ICH-S4 (Duration of Chronic Toxicity Testing in Animals), and ICH-S8 (Immunotoxicity Studies for Human Pharmaceuticals). Animals were 2–4 years old at injection, mixed sex (2M/2F per cohort), and were of Asian origin. Lumbar punctures were performed on days 1, 29, 57 and 85. The procedure was performed fasting under ketamine/medetomidine anesthesia with a pencil-point pediatric needle at a position between L2 and L6. First, ≥0.5 ml of CSF was collected, then, 20 mg ASO was delivered in a 1 ml volume of artificial CSF (aCSF) injected over 1 minute, followed by a flush of 0.25 ml aCSF. 15 minutes after the procedure, animals were awakened with atipamezole. CSF was ejected into Protein LoBind tubes and snap frozen in liquid nitrogen. The CSF samples analyzed here were collected at day 85, just prior to the fourth dose, while brain tissues were collected at day 92. Because the majority of CSF volume was used for regulated studies, the aliquots available for analysis in this study varied from 120–300 μl and 0.03% CHAPS was added only after freeze/thaw; these pre-analytical factors likely contribute additional variability between samples (35).

### Tissue dissection

For mouse brains, cryostat (Leica CM3050 S) dissection was performed as described (36): after storage in optimal cutting temperature (O.C.T.) compound (Tissue-Tek 4583) at -80°C, mouse brains were mounted by the frontal cortex onto cryostat chucks with O.C.T. leaving the entire posterior half of the brain exposed. A ∼2.5 mg piece of tissue was then excised using a pre-chilled ophthalmic microscalpel (Feather *P*-715) and placed into a pre-chilled PCR tube. For mice, a piece of somatosensory cortex was used for snRNA-seq, while an adjacent piece of visual cortex was used for bulk qPCR; thalamus was cut along the fiber tract and the dorsal half was used for snRNA-seq while the ventral half was used for qPCR; cerebellum was cut through the ansiform lobule and a piece of simple/ansiform lobule was used for snRNA-seq while a piece of ansiform/paramedian lobule was used for qPCR. Cynomolgus brains were coronally sectioned at a thickness of 4 mm, and cylindrical tissue punches of 2 mm diameter were taken for RNA analysis and of 6 mm diameter for protein analysis. The 2 mm diameter by 4 mm length cylindrical tissue punch was then sectioned lengthwise into quarters on the cryostat and one quarter was used for single cell analysis. From the most rostral section containing frontal cortex, punches were taken from middle frontal gyrus across all histological cortical layers. From section 13–14, where both cerebellum and medulla are visible, punches were taken from the posterior lobe of the cerebellum across the granular, ganglionic and molecular layers.

### Bulk tissue qPCR

Tissue pieces dissected on the cryostat were placed in RNAlater-ICE (Invitrogen AM7030) and allowed to thaw overnight at -20°C. Once samples were thawed, tissue was homogenized in 1 ml QIAzol lysis reagent, using 3 × 40 s pulses on a Bertin MiniLys homogenizer in 7 ml tubes pre-loaded with zirconium oxide beads (Precellys CK14, Bertin KT039611307.7/P000940-LYSK0-A). RNA was isolated from homogenate using RNeasy Lipid Tissue Mini Kit (Qiagen 74804) per the manufacturer protocol. RNA was eluted with 40 μl RNase-free water. RT-PCR samples were prepared using Taqman 1-Step RT-PCR master mix (Invitrogen) and Taqman gene expression assays (Invitrogen) for mouse *Prnp* (Mm00448389_m1; spanning exons 1–2) and mouse *Tbp* (Mm00446971_m1) and for cynomolgus *TBP* (Mf04357804_m1). The following gene-specific primer-probe sets were custom ordered from IDT: *Malat1* (mouse), Forward: AGGCGGGCAGCTAAGGA, Reverse: CCCCACTGTAGCATCACATCA, Probe: TTCCTCTGCCGGTCCCTCGAAAG; *PRNP* (cynomolgus; spanning intron 1–exon 2), Forward: CCTCTCCTCACGACCGA, Reverse: CCCAGTGTTCCATCCTCCA, Probe: CCACAAAGAGAACCAGCATCCAGCA. Samples were run on a QuantStudio 7 Flex system (Applied Biosystems) using manufacturer's recommended cycling conditions. Each biological sample was run in duplicate and the level of all targets were determined by ΔΔCt whereby results were first normalized to the housekeeping gene *Tbp* and then to PBS- or aCSF-treated animals.

### Single cell sequencing

After cryostat dissection, samples were batched in groups of eight, chosen to include treated and control animals in every run. Single nucleus suspensions were prepared as described (37,38). Briefly: tissue samples were triturated, by pipetting, in an extraction buffer containing Kollidon VA64, Triton X-100, bovine serum albumin, and RNase inhibitor, then passed through a 26-gauge needle, washed and pelleted, then passed through a cell strainer. Nuclei positive for DAPI signal were isolated by fluorescence-activated cell sorting with a Sony SH800 or MA900 calibrated with a 70 μm chip, with a 405 nm excitation laser and light collected with a 425–475 nm filter. Sorted nuclei were counted using a Fuchs-Rosenthal C-Chip hemocytometer and a hand tally counter. A volume chosen to target 17 000 nuclei was submitted to the Broad Institute's Genomics Platform, where 10X library construction (3' V3.1 NextGEM with Dual Indexing) was performed according to manufacturer instructions (39). Libraries were sequenced on an Illumina Novaseq 6000 S2 for 100 cycles.

### Data processing and analysis

Raw binary base call (BCL) files were synced to Google Cloud and analyzed on Terra.bio. Cumulus (40) Cell Ranger (41) 6.0.1 (cellranger_workflow v28) was employed, with flags –include_introns and –secondary set to true, to process BCL files into unique molecular identifier (UMI) count matrices for each individual sample. Mouse samples were aligned to Cell Ranger reference package mm10-2020-A and cynomolgus samples were aligned to a custom Cell Ranger reference made from Ensembl *Macaca fascicularis* 6.0 (release 108). Matrices were then aggregated using Cell Ranger 7.0.1 (aggr with the –normalize flag set to none) to yield one UMI count matrix per species and brain region. Statistical analyses and data visualization were conducted using custom scripts in R 4.2.0.

### Cell type assignment

Aggregated count matrices were examined using Loupe Browser. Viewing cells in 2-dimensional uniform manifold approximation and projection (UMAP) (42) space, we looked for cell type markers established or validated in several prior single-cell studies (36,43–49). Clusters corresponding to empty droplets, doublets, debris, or mitochondria were flagged and removed based on low UMI or unique gene count, low percentage intronic reads, lack of obvious differentially expressed genes, high expression of mitochondrial genes, or location between two other clusters and expression of markers of each. Assignments were then validated by generating dot plots in Seurat V4 (50) in R. For cortical excitatory and inhibitory neurons in 3 week post-dose animals, a list of barcodes was exported from R and reclustered in Loupe Browser.

### Pharmacokinetic studies

Quantification of ASO N in NHP tissue was performed as described (51). Briefly, tissue samples were weighed, homogenized, and extracted first using a liquid-liquid extraction (LLE) with ammonium hydroxide and phenol:chloroform:isoamyl alcohol (25:24:1), followed by a solid phase extraction (SPE) using a 96-well Strata X packed plate (Phenomonex), followed by a pass through using a Protein Precipitation Plate (Phenomonex). Eluates were dried down under nitrogen at 50°C before reconstituting with 100 μl water containing 100 μM EDTA. Samples were then analyzed by ion-pairing (IP) LCMS/MS with an Agilent 6460 LCMS/MS system (Agilent), using an ACQUITY UPLC OST C18 column (Waters) heated to 55°C with a flow rate of 0.3 ml/min. The column was equilibrated with 400 mM HFIP/15 mM TEA in water. A gradient from 10 to 40% MeOH over 6 min was used to elute ASO N. All mass measurements were made on-line with MRM transitions of *m*/*z* 881.6 and 773.2 both with a product ion of 94.8 for ASO N, and the internal standard, respectively. Mass spectra were obtained using a spray voltage of –1500 V, a nebulizer gas flow of 25 psig, a sheath gas flow rate of 12 l/min at 350°C, a drying gas flow rate of 5 l/min at 350°C, and a capillary voltage of –3750 V. Chromatograms were analyzed using Agilent Mass Hunter software. ASO N concentration was determined from its calibration curve with dynamic range 0.035 μg/g (0.05 μM) to 176.95 μg/g (25.0 μM).

### Statistics

For each combination of brain region, timepoint, and treatment condition, snRNA-seq data were grouped by animal and cell type and the sum of target UMIs and total UMIs was calculated. A negative binomial model was fit to the resulting data, with target RNA UMIs as the dependent variable; cell type and a cell type-treatment interaction term as the dependent variables, and total UMIs as the offset. This utilized the MASS package in R, with the call: glm.nb(target_umi ∼ celltype + celltype:treatment +offset(log(total_umi))). This returns coefficients in natural logarithm space. For the ASO-treated conditions, the coefficient for each cell type-treatment interaction term coefficient was then exponentiated to yield the mean estimate of the residual target RNA in that cell type. The 95% confidence interval was defined as that mean estimate ±1.96 of the standard errors returned by the model. Each individual animal's point estimate of residual target RNA in each cell type was obtained by adding the residual from the model to the cell type-treatment coefficient, and then exponentiating. To account for the different abundance of different cell types, which impacts the size of our confidence intervals on target knockdown, we used weighted Pearson's correlations (wtd.cor from the weights package in R) to test candidate variables and weighted standard deviations (square root of wtd.var from the Hmisc package in R) to evaluate the variability in target engagement between cell types within different brain regions. Throughout, all error bars and shaded areas in figures represent 95% confidence intervals. P values less than 0.05 were considered nominally significant.

## RESULTS

### Generation and cell type classification of single nucleus transcriptomes

**Figure 1.**
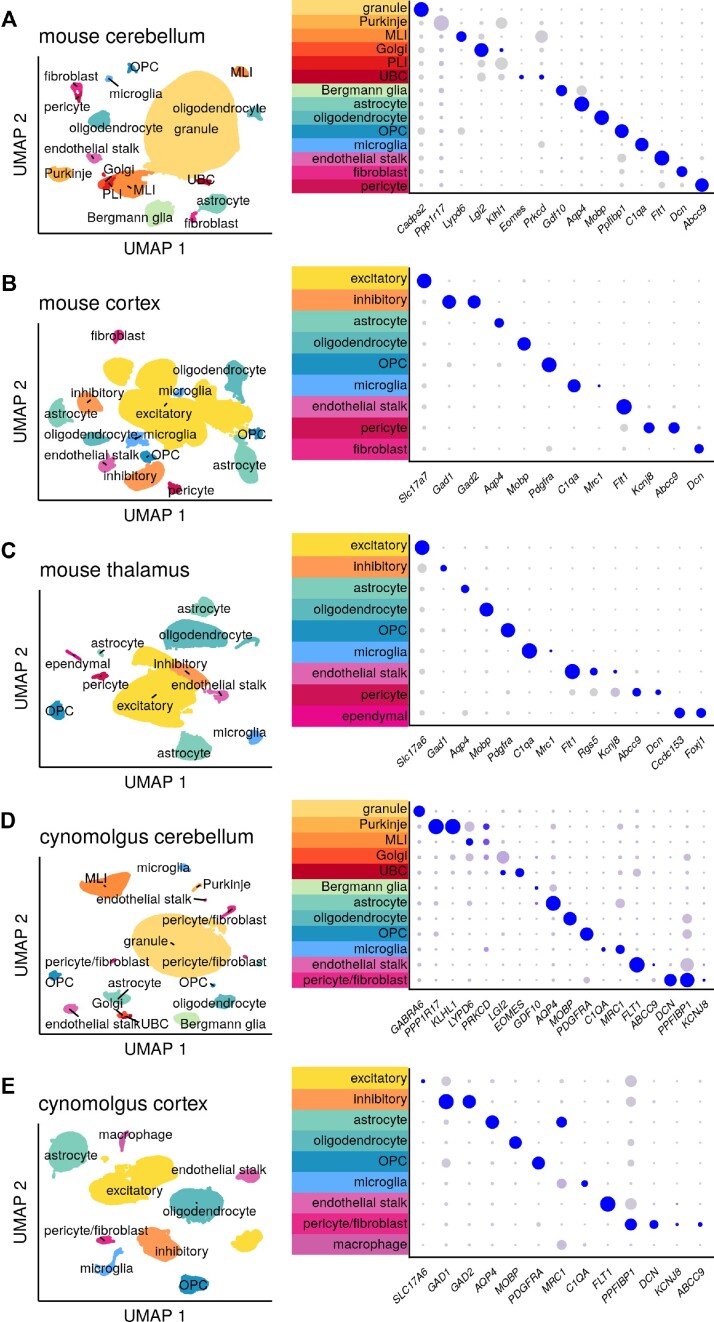
Clustering and assignment of brain cell types. Single-cell gene expression profiles projected into two dimensions using Uniform Manifold Approximation and Projection (42) (UMAP) and characterized using dot plots. In dot plots, gray to blue color gradient represents higher expression while small to large dot size gradient represents broader expression. Thus, a large blue dot indicates a marker widely and highly expressed by cells within the indicated cluster; a small gray dot indicates little to no expression by those cells. A small blue dot can indicate a marker highly expressed by only a subset of cells within the cluster, while a large gray dot can indicate a marker broadly but lowly expressed. MLI = molecular layer interneuron, UBC = unipolar brush cell, PLI = Purkinje layer interneuron, OPC = oligodendrocyte progenitor cell. For breakdown by weeks post-dose and active/inactive treatment group see Supplementary Figure S1.

We selected 4 previously characterized ASOs: 2 *Prnp* ASOs that extend survival in prion-infected mice (32,52), 1 *Malat1* ASO with extensive pharmacology data (21), and 1 human *PRNP* ASO sequence-matched in macaques (53) (Table 1). We analyzed a total of 78 single nucleus transcriptomes from tissues of mice and macaques treated with these ASOs or with vehicle (Supplementary Table S1-2) totaling 598066 single nuclei. Samples averaged 532 million reads mapping to 7667 cells and yielding 7650 unique molecular identifiers (UMIs) per cell, corresponding to a median of 3108 detected genes per cell (Supplementary Table S1). Transcriptomes were aggregated by species and brain region yielding five count matrices. Distinct clusters were assigned cell types using established markers (Figure 1, Supplementary Figure S1). The number of nuclei per cell type correlated with reference datasets (36,44,54) (Supplementary Figure S2, Supplementary Tables S3–S5).

**Table 1.** Compounds used in this study. Color code for ASO chemical modifications: black = unmodified deoxyribose (2′H; DNA). orange = 2′ methoxyethyl (MOE). blue = 2′-4′ constrained ethyl (cET). Unmarked backbone linkages = phosphorothioate (PS); linkages marked with o= normal phosphodiester (PO). mC = 5-methylcytosine

ASO	sequence and chemistry	target	ref
ASO 1	mCToAoTTTAATGTmCAoGoTmCT	mouse *Prnp* 3'UTR	(32,52)
ASO 6	mCToTomCoTATTTAATGTmCAoGoTmCT	mouse *Prnp* 3' UTR	(32)
*Malat1* ASO	GComCoAoGoGmCTGGTTATGAomCoTmCA	mouse/NHP *Malat1*	(21)
ASO N	GTomCoAoToAoATTTTmCTTAGmCoTAmC	human/NHP *PRNP* intron	(53)

### Distribution of ASO activity at the single cell level

**Figure 2.**
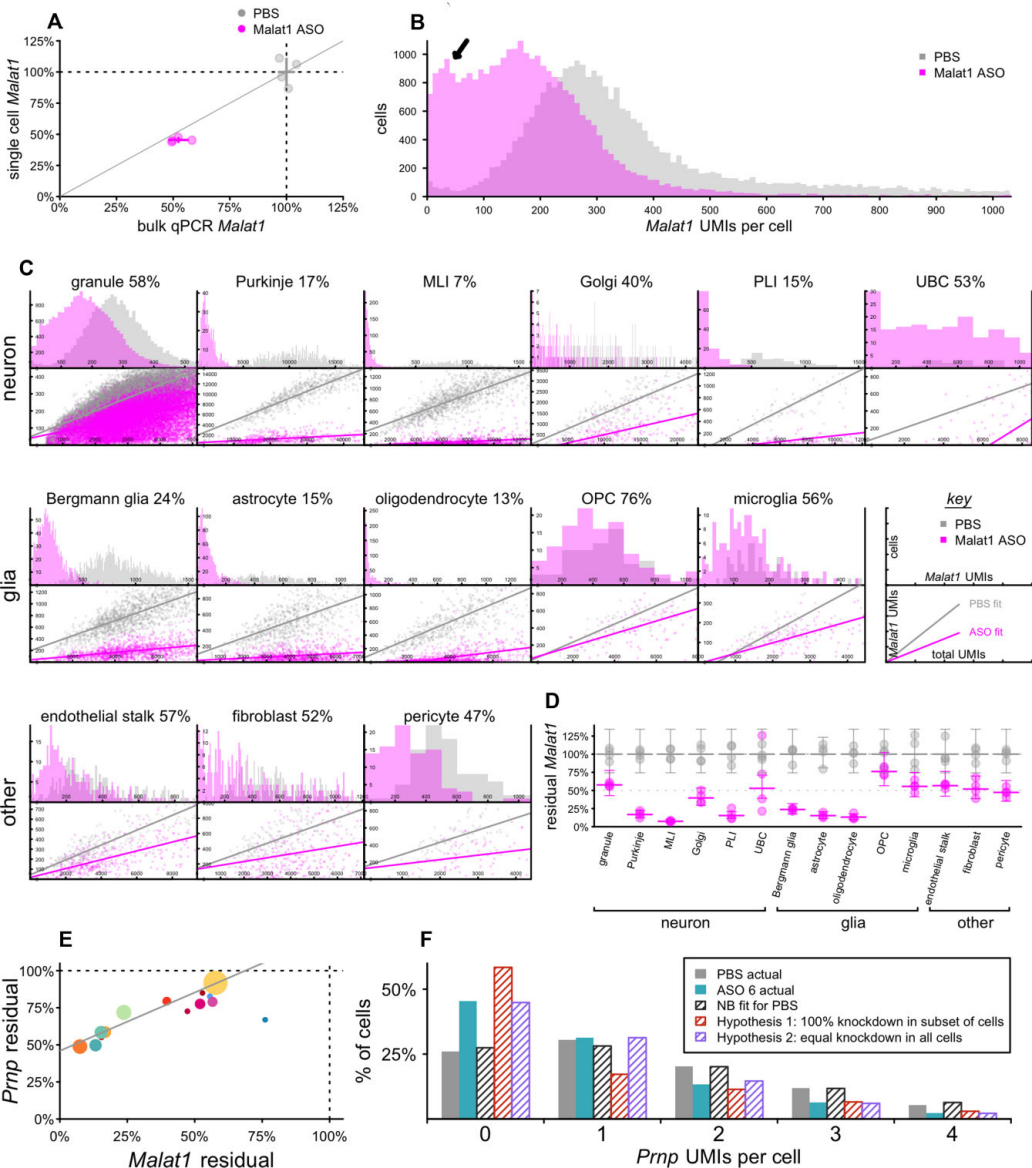
Single-cell distribution of ASO activity in mouse cerebellum at 12 weeks post-dose. Mice received 50 μg Malat1 ASO (*N* = 4), 500 μg ASO 6 (*N* = 4), or PBS (*N* = 4) ICV and cerebella were harvested 12 weeks later. (**A**) Malat1 knockdown after Malat1 ASO treatment, assessed by bulk qPCR (x axis) versus aggregation of single cell sequencing regardless of cell type (y axis), for individual animals (points) and groups (crosshairs indicate means and 95% confidence intervals). (**B**) Histogram of the number of Malat1 UMIs per single cell across all samples. Arrow indicates a second peak observed only for treated animals. (**C**) Breakdown across 14 cell types. Top panels are histograms of single cells as in (B), but broken down by cell type. Bottom panels are scatterplots showing total UMIs per single cell versus Malat1 UMIs per cell and best fits by linear regression (Supplementary Table S12). Percentages indicate residual Malat1. Note the ‘key’ panels at the far right of the middle row. (**D**) Negative binomial (NB) modeling of single cell data as point estimates of knockdown for each animal and each cell type (points) and means and 95% confidence intervals (bars) for each treatment group and cell type. (**E**) Correlation across cell types of Prnp knockdown by ASO 6 and Malat1 knockdown by Malat1 ASO. Each point is a cell type, colors are from Figure 1A, and point sizes are logarithmically scaled with number of cells sequenced. (**F**) Histogram of Prnp UMIs per single cell in astrocytes for PBS (gray) and ASO 6-treated animals (cyan). Shaded gray bars indicate the distribution predicted by a NB model fit to the PBS data. Shaded red bars indicate the distribution if the observed 56% residual Prnp RNA in astrocytes corresponded to 56% of cells following the original NB distribution and 44% being set to zero. Shaded cyan bars indicate the distribution if the observed 56% residual corresponded to the NB parameter mu being reduced by 44%.

The long non-coding RNA *Malat1* is a valuable model target for single cell assessment of ASO activity because it is highly expressed, accounting for 11.4% of all UMIs in our mouse transcriptomes, and because a potent and well-characterized tool ASO against *Malat1* is available (21). The high expression means that *Malat1* averages hundreds of UMIs per individual cell—an asset when evaluating knockdown in single cells—whereas most genes exhibit Poisson distributions with many zeroes (Supplementary Table S11) (55). Considering the *Malat1* ASO’s median effective dose (ED_50_) of ∼50 μg in cerebellum (21) and the prior evidence for some difference in activity between cerebellar cell types (21,27), we examined *Malat1* knockdown in cerebellum at 12 weeks after a single 50 μg ICV dose of *Malat1* ASO. Aggregation of single nucleus sequencing data across all mouse cerebellar nuclei indicated 45.4% residual *Malat1*, close to the 52.4% residual detected in an adjacent piece of cerebellar tissue analyzed by bulk qPCR (Figure 2A; Supplementary Table S6-S7). When *Malat1* UMIs per cell were visualized as a histogram (Figure 2B), the median cell possessed 360 *Malat1* UMIs in PBS-treated animals. ASO treatment yielded a bimodal histogram, with a main peak at 166 UMIs but a second peak at ∼35 UMIs (black arrow, Figure 2B) indicating deeper knockdown in a subpopulation of cells. Across 14 different cell types (Figure 2C), residual *Malat1* ranged from 7% to 76%, while each individual histogram appeared unimodal. This suggested that the bimodality in the histogram for bulk tissue (Figure 2B) is due to differences in knockdown in different cell types. Scatterplots of *Malat1* UMIs vs. total UMIs indicated that the width of the distributions in these histograms is largely due to differences in total UMIs per cell. All of these observations were consistent with broad knockdown in every detected cell. Fitting residual *Malat1* in each cell type in each sample with a negative binomial model (see Materials and Methods) confirmed substantial differences in knockdown between cell types (Figure 2D; Supplementary Tables S8–S10).

snRNA-seq inherently yields low sequencing coverage in any one nucleus, meaning that most genes are not detected in most nuclei, even where they are expressed (Supplementary Table S11). Unlike the highly expressed *Malat1*, most potential ASO targets will have UMI counts that are Poisson or negative binomial distributed in single nuclei data. For instance, *Prnp* averaged just 0.85 UMIs/cell in the cerebella of PBS-treated animals. Nevertheless, when the data from 12 weeks after a single 500 μg dose of ASO 6, were fit to the same negative binomial model as *Malat1*, ASO 6 displayed a highly similar pattern of activity across cerebellar cell types (rho = 0.96, *P* < 3.9e–8, weighted Pearson's; Figure 2E; Supplementary Table S10).

Despite lower basal expression, we posited that examination of UMI/cell histograms for *Prnp* could reveal information about the distribution of drug activity across single cells. As an example, we compared histograms for astrocytes, which had 56% residual *Prnp* in ASO 6-treated animals, versus three models. A negative binomial model fit to the PBS-treated animals mirrored those animals' actual distribution almost perfectly. Lowering *Prnp* to 56% residual by setting 44% of astrocytes' *Prnp* counts to zero would have yielded a histogram with far more zeroes, and fewer ones, than the observed distribution in ASO 6-treated animals. In contrast, lowering *Prnp* to 56% residual by lowering the negative binomial parameter *mu* by 44%, corresponding to equal knockdown in all cells, yielded a distribution nearly identical to that in ASO-treated animals (Figure 2F; Supplementary Table S13). Thus, for *Prnp* as for *Malat1*, bulk tissue knockdown appears to arise from broad knockdown in all cells, albeit with a stereotypical pattern of differences across distinct cell types.

### ASO activity across regions and cell types in the mouse brain

**Figure 3.**
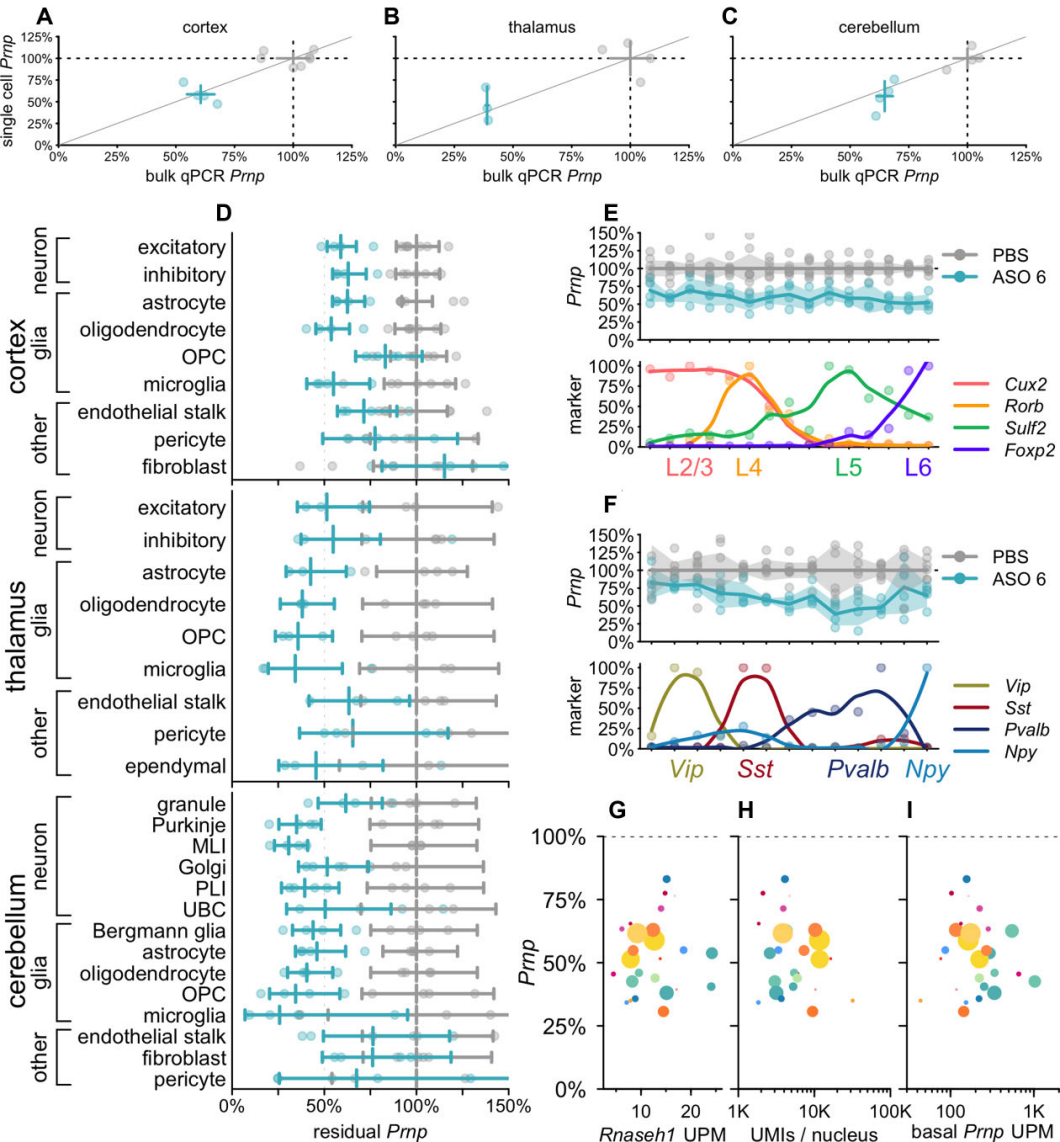
Atlas of ASO activity across cell types in mouse brain at 3 weeks post-dose. Mice received a single 500 μg dose of ASO 6 or PBS and were harvested 3 weeks later. *N* = 4 ASO 6 versus *N* = 7 PBS for cortex, *N* = 3 ASO 6 versus *N* = 4 PBS for thalamus, *N* = 4 ASO 6 versus *N* = 4 for cerebellum. (**A–C)** Concordance of bulk qPCR (x axis) and single cell (y axis) measurement of total Prnp knockdown in cortex (A), thalamus (B) and cerebellum (C). Points are individual animals, crosshairs are 95% confidence intervals on both dimensions. (**D**) Residual Prnp expression across cell types in three brain regions. Error bars are 95% confidence intervals of the mean. (**E**) Cortical excitatory neurons from panel A were reclustered into 15 clusters ranked by expression of four excitatory layer markers. Residual Prnp (top) is shown as individual animals (points), means (lines), and 95% confidence intervals of the mean (shaded areas). Each marker's expression (bottom) is normalized to the cluster with the highest expression; points are normalized values and curves are loess fits. (**F**) Cortical inhibitory neurons from panel A, reclustered and plotted as in panel B. (**G–I)** Scatterplots of residual Prnp expression (y axis) versus candidate covariates (x axes). Each point represents a region and cell type combination from panel D (cortical fibroblasts, the only cell type with nominally >100% residual expression, are not visible). Colors correspond to cell type colors in Figure 1, and dot sizes scale logarithmically with the number of cells sequenced. Candidate covariates examined are Rnaseh1 expression in UMIs per million (UPM; G), total UMIs per cell (H) and basal Prnp expression (UPM; I).

We assessed the profile of ASO target engagement across cell types in 3 brain regions in mice at 3 weeks post-dose with *Prnp* ASO 6 (Figure 3). Because this tool compound is less potent than the *Malat1* ASO, we used a 500 μg dose, which modifies prion disease in mice (32,52) and lowers whole hemisphere PrP to an estimated 56% residual after 4 weeks (56). Whereas *Malat1* localizes to the nucleus (57), *Prnp* is a protein-coding gene whose mRNA reaches the cytosol, and ASO 6 targets the *Prnp* 3'UTR, so cytosolic activity is possible. Nonetheless, we found that the value of residual *Prnp* obtained by snRNA-seq, which will detect nuclear ASO activity only, agreed closely with the value obtained by bulk tissue qPCR, which used exon junction-spanning primers and therefore will only detect mature mRNA (Figure 3A–C).

Breakdown of single cell data by cell type showed broad target engagement across cell types including diverse types of neurons and glia (Figure 3D). As with the *Malat1* ASO (Figure 2), cell type differences were relatively pronounced in the cerebellar neurons, where knockdown was deeper in Purkinje cells and MLI than in granule cells. Across regions in ASO-treated animals, endothelial stalk cells, pericytes, and fibroblasts generally had both the highest residual *Prnp* and the lowest count of cells sequenced, giving rise to wide confidence intervals that overlapped the PBS-treated animals. Nonetheless, point estimates for these cells generally suggested some target engagement, with the possible exception of cortical fibroblasts. To further examine the profile of knockdown among neuronal subtypes, we reclustered cortical excitatory (Figure 3E; Supplementary Table S14) and inhibitory (Figure 3F; Supplementary Table S15) neurons and ordered them by relative expression of excitatory layer or inhibitory subtype markers. Target engagement appeared similar across all layers of excitatory neurons (Figure 3E). Knockdown appeared possibly deeper in *Pvalb*-expressing than in *Vip*-expressing inhibitory neurons, but again, target engagement was observed across all subtypes (Figure 3F). Across all regions, differential knockdown across cell types was not explained by *Rnaseh1* expression, which varied little (*P* = 0.61, weighted Pearson's correlation; Figure 3G), nor total UMIs per nucleus, a potential proxy for cell size (36), nor basal *Prnp* expression (*P* = 0.64 and *P* = 0.08, weighted Pearson's correlation; Figure 3H-I; see Discussion).

### Potency and duration of action across ASO chemistries

**Figure 4.**
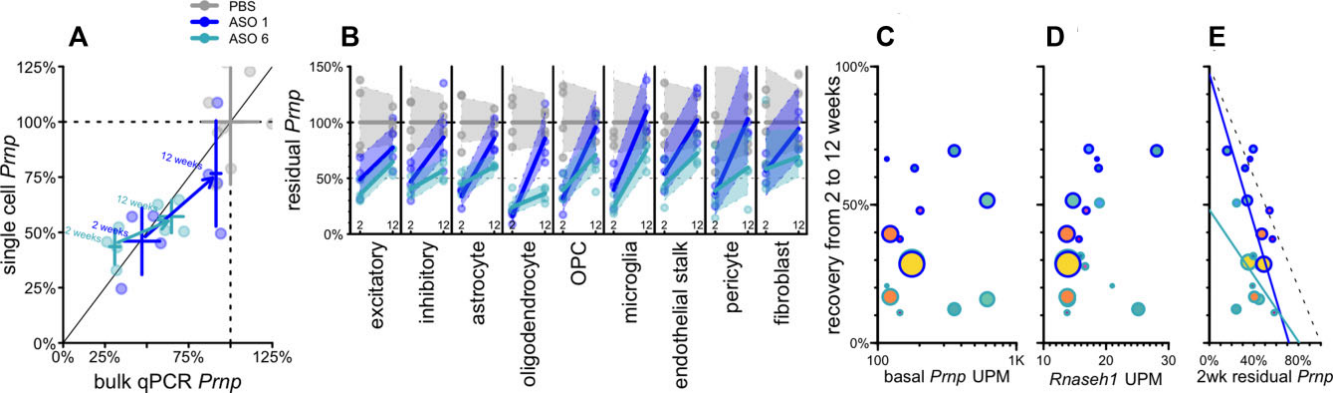
Duration of activity across different ASO chemistries in mouse cortex from 2 to 12 weeks post-dose. Mice received PBS (*N* = 4 per timepoint), 500 μg ASO 1 (*N* = 4 per timepoint), or 500 μg ASO 6 (*N* = 4 per timepoint) and cortex was harvested after 2 or 12 weeks. (**A**) Overall residual Prnp assessed by bulk qPCR (x axis) versus aggregate single cell data without regard to cell type (y axis). Points represent individual animals and crosshairs represent means and 95% confidence intervals on both axes. 2 week data (lower left) and 12 week data (upper right) are connected by arrows indicating washout for both ASO 1 (dark blue) and ASO 6 (cyan). (**B**) Washout from 2 weeks (left) to 12 weeks (right) for each cell type in mouse cortex for ASO 1 and ASO 6. Lines represent means and shaded areas represent 95% confidence intervals. (**C–E**) Scatterplots of candidate variables (x axis) basal Prnp expression (UMIs per million, UPM; C), Rnaseh1 expression (UPM; D), and 2 week residual Prnp (E) versus percentage points of recovery (washout at 12 versus 2 weeks, y axis) for each cell type (cell types are points, sized logarithmically by number of cells) for both ASO 1 (dark blue outlines) and ASO 6 (cyan outlines), with symbol fill color indicating cell type (color scheme according to Figure 1B).

Gapmer ASOs currently in clinical trials are 2'MOE gapmers similar to ASO 6, but improved chemical modifications of ASOs are a highly active area of research (58,59), prompting us to investigate the cell type profile of an ASO incorporating 2'–4' constrained ethyl (cEt) modifications (60). *Prnp* ASO 1 (Table 1), a mixed 2'MOE/cEt oligonucleotide, targets the same site as ASO 6 and is effective in prion-infected mice (32,52). We evaluated the activity of ASO 1 and ASO 6 in mouse cortex at both 2 and 12 weeks after a single 500 μg bolus dose (Figure 4). Again, single cell and bulk qPCR measurements of overall knockdown concurred (Figure 4A). ASO 1 had a shorter duration of action than ASO 6, with residual target rising from 47% to 91% of saline controls (by bulk qPCR) residual, a 44% recovery, versus 31% to 65%, a 34% recovery, for ASO 6 (Figure 4A; Supplementary Table S7). Each compound provided substantial knockdown at 2 weeks across all cell types detected, and each exhibited marked differences across cell types in the rate of recovery (Figure 4B; Supplementary Table S16). For example, for both ASOs, microglia exhibited the most complete recovery of any cell type (+51% for ASO 6 and +70% for ASO 1), while excitatory neurons were comparatively steady (+29% for both; Figure 4B).

Across cell types for both compounds, neither basal *Prnp* expression (RPM in PBS-treated animals) nor *Rnaseh1* expression showed any correlation with washout between 2 and 12 weeks (*P* = 0.78 and *P* = 0.12, weighted Pearson's correlation; Figure 4C-D). The depth of target suppression at 2 weeks post-dose, however, showed an inverse correlation with recovery by 12 weeks which was significant for ASO 1 (rho = -0.84, *P* = 0.0043, weighted Pearson's correlation) and directionally consistent for ASO 6 (rho = -0.41, *P* = 0.27, weighted Pearson's correlation; Figure 4E; see Discussion).

### Cell type profile and biomarker impact in non-human primates

**Figure 5.**
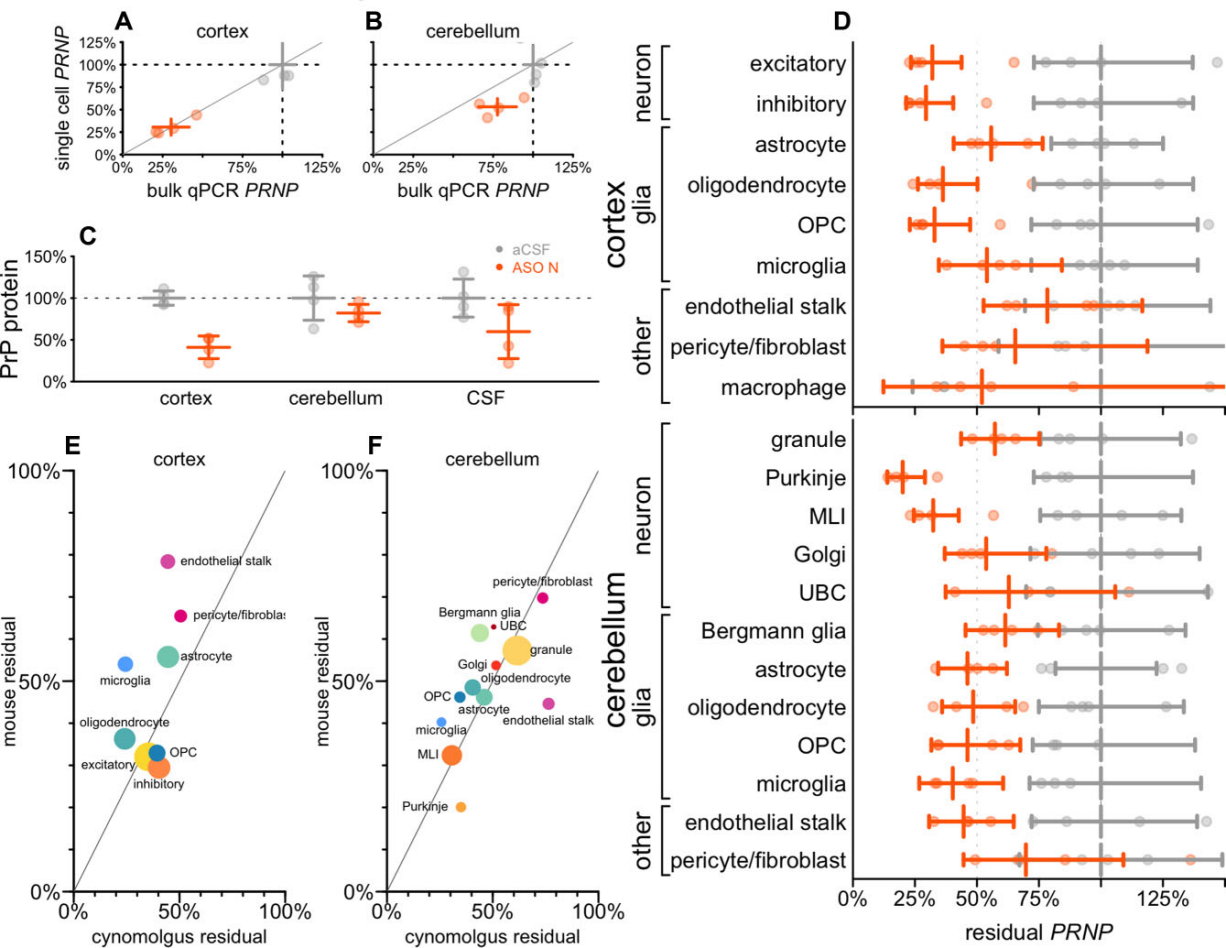
Cell type activity distribution and biomarker response in non-human primates. Cynomolgus macaques received 20 mg ASO N (*N* = 4) or aCSF (*N* = 4) at weeks 0, 4, 8 and 12, and cortex and cerebellum were evaluated at week 13. (**A, B**) PRNP knockdown assessed by bulk qPCR (x axis) versus aggregation of single cell sequencing regardless of cell type (y axis), for individual animals (points) and groups (crosshairs indicate means and 95% confidence intervals) in cortex (A) and cerebellum (B). (**C**) PrP protein measured by in-house ELISA (56) in brain parenchyma (cortex, cerebellum) and in cerebrospinal fluid (CSF). (**D**) Residual PRNP by cell type in cortex and cerebellum. (**E, F**) Scatterplot of cynomolgus 13-week residual PRNP by cell type (x axis) versus mouse residual Prnp at 2 weeks after a single dose of ASO 6 (data from Figure 3A) in cortex (E) and cerebellum (F). The data point for pericyte/fibroblast reflects the weighted average of residual Prnp in these two cell populations in mouse. Each point is a cell type, sized logarithmically by total number of cells (cynomolgus + mouse datasets) and colored as in Figure 1.

We examined tissue from cynomolgus macaques that received ASO N. In addition to permitting us to examine ASO activity in a larger brain, the macaques also differed from our mice in being dosed intrathecally (IT) rather than ICV, and receiving 4 repeat doses at 4-week intervals. Both cortex and cerebellum exhibited substantial drug accumulation (Supplementary Figure S3, Supplementary Table S17-S18). In cortex, bulk residual *PRNP* measured by snRNA-seq again mirrored that by qPCR (Figure 5A), although in cerebellum, knockdown measured by snRNA-seq appeared slightly deeper (Figure 5B). Residual PrP protein level quantified by ELISA (56) in ASO N-treated animals was 41% in cortex, 82% in cerebellum and 60% in CSF (Figure 5C; Supplementary Tables S19 and S20). Target engagement was broadly observed across all detected cell types in both cortex and cerebellum (Figure 5D; Supplementary Table S10). In cortex, knockdown was deepest in neurons and weakest in endothelial stalk and pericytes/fibroblasts. In cerebellum, knockdown was deepest in Purkinje cells and molecular layer interneurons (MLIs) and weakest in pericytes/fibroblasts. Because these tissues were obtained just 1 week after the animals' final dose of ASO, we compared the cell type profile of target engagement in macaques to that observed in mice 2 weeks after a single dose of ASO 6 (Figure 5E, F). The two datasets shared robust knockdown in MLI, Purkinje, and cortical neurons and relatively limited knockdown in pericytes/fibroblasts. Correlation of knockdown across cell types was positive, though significant only in cerebellum (rho = 0.40 and 0.80, *P* = 0.33 and 0.0019 for cortex and cerebellum respectively, weighted Pearson's).

### Comparison of cell type specificity across paradigms

**Figure 6.**
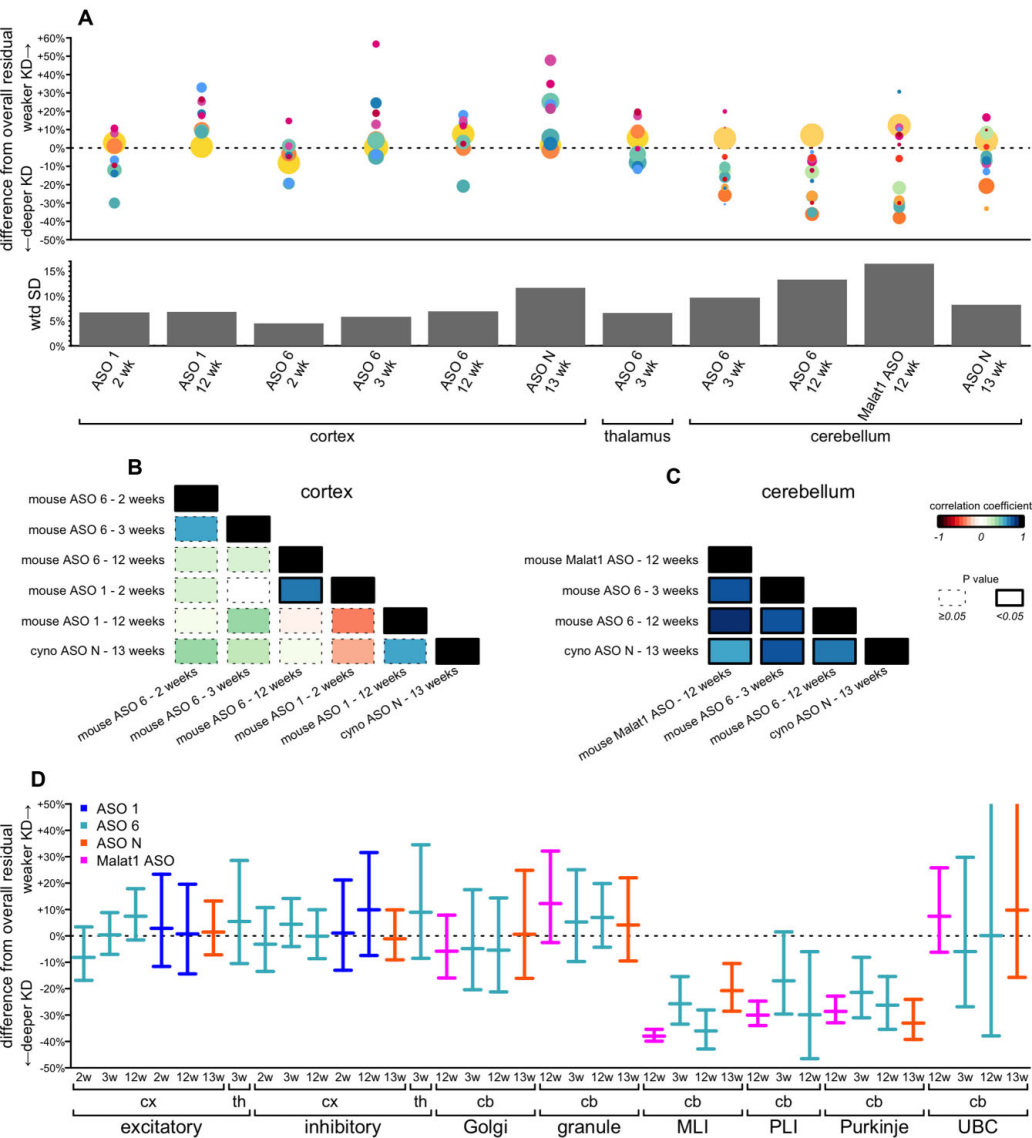
Comparison of cell type target engagement profiles across all conditions examined. (**A**) Top: differences from overall residual (each cell type's residual target minus the overall residual target quantified from single-nucleus data; y axis) for every combination of tissue, ASO, timepoint, and species (shared x axis). Each point is a cell type, sized logarithmically by number of cells and colored as in Figure 1. Bottom: weighted standard deviations (weighted by number of cells) in percentage points of residual target (y axis) for each condition (shared x axis). (**B, C**) Correlograms (weighted Pearson's correlations) between every pair of datasets for cortex (B) and cerebellum (C). Colors represent the value of the correlation coefficient rho and the outline represents the nominal P value, see legend at far right. (**D**) Differences from overall residual for all neuronal subtypes in all conditions. Error bars are 95% confidence intervals from the negative binomial models for the subtype-specific residuals.

To ask how broadly the profile of activity across cell types was shared among all our datasets, we defined a difference from overall residual as a cell type's residual target RNA, expressed as a percentage of control animals, minus the overall residual target RNA across all cell types (Supplementary Table S21). In cortex, the most abundant cell types, chiefly neurons, clustered near 0% (excitatory neurons, mean + 1%, inhibitory neurons, mean + 2%), reflecting the bulk tissue closely; outliers were rarer cell types with wider confidence intervals (Figure 6A). In cerebellum, however, granule cells (mean + 7%) differed considerably from the next two most abundant cell types, MLIs (–30%) and Bergmann glia (–10%). Variability across cell types was lower in cortex and thalamus (mean weighted standard deviation 7% for both) than in cerebellum (mean weighted standard deviation 12%; Figure 6A). Accordingly, we observed mostly positive but non-significant correlations between pairs of cortex datasets (Figure 6B; Supplementary Table S21), whereas all correlations were strongly positive and significant in cerebellum (Figure 6C; Supplementary Table S22). Neurons were generally either close to the bulk tissue residual (worst case, +12% difference for granule cells in *Malat1* ASO-treated mouse cerebellum at 12 weeks) or exhibited much deeper target engagement (-38% for MLIs in *Malat1* ASO-treated mouse cerebellum at 12 weeks; Figure 6D; Supplementary Table S23). We did not observe any conditions in which any population of neurons exhibited dramatically weaker knockdown than the bulk tissue.

## DISCUSSION

Here we deployed single nucleus transcriptomics to quantify ASO target engagement in the CNS. We observed a broad distribution of activity across individual cells, but with differences in activity and in duration of action between cell types. The profile of activity across cell types was largely shared across different ASOs examined and between mouse and NHP.

### Distribution of ASO activity across individual cells

Theoretically, 50% knockdown in bulk tissue could result, in the most extreme cases, from either 100% knockdown in half of cells or 50% knockdown in all cells. A longstanding question is where the activity of CNS ASOs falls on this spectrum.

By examining the distribution of target RNA counts per cell in single nucleus sequencing data, we provide evidence of ASO activity in every single cell in a bulk tissue, albeit with differences in degree between cell types. This should be expected based on the number of drug molecules contained in a dose of ASO. For a mouse with ∼10^8^ brain cells (61,62), a 50 μg dose of a ∼7 kDa ASO, or ∼4 × 10^15^ ASO molecules, is >10^7^ molecules per brain cell. Only a small minority of ASO molecules are believed to undergo productive uptake (3), but even if this figure is 1%, then 10^5^ productive ASO molecules per brain cell is a sufficient number that it is unlikely that any cells would avoid ASO activity simply by chance. Of course, there may be cells in deep brain structures lacking any appreciable ASO activity due to limited drug distribution (21); we only analyzed tissues with robust target engagement at the bulk level. Under this precondition, ASO activity appears very broadly distributed across individual cells.

This property of ASOs could prove markedly different from some gene therapy approaches to CNS diseases. In mice, engineered viral vectors for gene delivery may transduce ∼50% of CNS neurons (63), and DNA-targeted therapeutics, with only 2 targets per cell, could provide nearly complete target suppression in those cells that are transduced. If so, modalities exhibiting similar levels of bulk target engagement could reflect rather distinct distributions at the single-cell level. These contrasting profiles might in turn present opposing challenges and opportunities for different targets.

### Differences in activity and duration of action between cell types

We observed differences in target engagement and in duration of action between cell types. There exist multiple possible mechanistic explanations for these differences. Distinct cell types could diverge at various steps in ASOs' cellular pathway (3) including differences in gross uptake, in the proportion of productive uptake, in the kinetics of endosomal escape, in the rate of RNase H1 cleavage, in the rate of release from the cell, in activity of nucleases that degrade the ASO, or in the presence or absence of cell division diluting out the ASO.

While the short duration of action in microglia would at first glance appear consistent with a role for dilution by cell division, the estimated microglial turnover rate (median lifetime >15 months (64)) is too slow to appreciably dilute ASO. Moreover, we observed comparatively weak knockdown in cells of the vasculature—fibroblasts, pericytes, and endothelial cells—which are described as largely quiescent in the adult brain (65).

Histological analysis of ASO-treated brain tissue indicates that the difference in ASO activity between granule and Purkinje cells may be due to total ASO uptake (21,27). It may be, however, that not all differences between cell types are explained simply by gross uptake. In lungs of mice treated intratracheally with divalent siRNA, fibroblasts exhibited deeper target engagement than other cell types despite lower drug accumulation (66); an LNA ASO was similarly most active in lung fibroblasts (31). In our dataset, across cell types in the mouse cortex, deeper initial target engagement at 2 weeks appeared to correlate with more washout by 12 weeks. This correlation is expected to some degree, because target expression after washout should never recover to >100% of the untreated condition, but may also suggest that deeper initial knockdown in some cell types does not necessarily indicate a longer-lasting endosomal repository of compound.

Our dataset is ill-suited to ask genome-wide questions such as which specific cell surface proteins are most important for uptake, because any two cell types differ in the expression of many markers, not just one, and in addition, the thousands of possible answers present a large multiple testing burden which cannot be overcome by analyzing the small number of distinct cell types detected here. None of the few specific hypotheses we tested appear to explain the cell type differences we observed. Cell size might be inversely related to the surface area to volume ratio, and thus to the amount of opportunity for cell surface protein binding, but UMIs/nucleus, a proxy for cell size (36), was not correlated with ASO activity in our dataset. RNase H1 expression varied little across cell types and neither RNase H1 nor target expression correlated with initial target engagement or washout. In fact, this should be expected based on the number of drug molecules per cell. PrP RNA expression is on the order of hundreds of transcripts per million (67), so a cell with 10^5^ mRNA molecules might have just tens of PrP mRNA molecules, not nearly enough to saturate 10^5^ productively uptaken ASO molecules.

### Limitations of this study

Our study has many limitations. The expense of single-cell sequencing limited us to small cohort sizes (usually *N* = 4). For some rarer cell types, just a handful of cells per sample were observed. Many steps including nuclei dissociation, flow cytometry, and library construction, can all yield variability in number of cells and number of sequencing reads per sample. All of these factors combine to make the confidence intervals on our estimates of knockdown in many cell types rather large.

Certain key observations replicate across our datasets—particularly the broadness of target engagement across cell types, with weaker knockdown in granule cells and deeper knockdown in Purkinje and interlayer neurons, and the generally weaker knockdown in cells of the vasculature. However, we studied only 3 brain regions, 4 ASOs, 2 targets, and 2 animal species, so it remains to be determined just how broadly these findings may generalize. We observed similar patterns of target engagement across cell types in mice treated with a high dose (500 μg) of a low potency ASO targeting *Prnp* and with a low dose (50 μg) of a high potency ASO targeting ubiquitously expressed *Malat1*, however, these experiments used different compounds against different targets, and it remains to be determined how the cell type specificity of ASOs differs as a function of dose response for a single compound.

Likewise, we observed duration of action differences in certain cell types between a 20-nucleotide 2’MOE gapmer ASO and a 17-nucleotide 2’MOE/cEt ASO, but without testing a more thorough battery of compounds, it is impossible to discern whether these differences are effects of chemical backbone, of length, or simply of random chance.

We lack any method of quantifying drug concentration in the same cells that are sequenced, so are unable to answer questions about the pharmacokinetic/pharmacodynamic relationship at the single-cell level.

Because we relied on purification of nuclei from frozen tissue, we were only able to measure target engagement in the nucleus. It is reassuring that snRNA-seq and qPCR generally agreed, but these analyses were necessarily performed on adjacent pieces of tissue, making it unclear whether their occasional divergence represents discordance between cytosolic and nuclear outcomes, or simply regional gradients in target engagement.

### Implications for preventive trials in prion disease

Pharmacologic interventions are seldom trialed in pre-symptomatic individuals at risk for neurodegenerative disease (68). Observing clinical endpoints in such individuals may require lengthy follow-up (69) or may be outright numerically infeasible (70). This has led to the suggestion that in prion disease, where the central role of PrP in disease is incontrovertible (71), the lowering of CSF PrP—a target engagement biomarker only—could serve as a primary endpoint in trials of at-risk individuals (72). This prospect demands that especially strong data from animal studies will be needed to certify the links between CSF PrP, target engagement in the disease-relevant cells, and disease modification (73).

In prion disease, the critical cells to engage are neurons. Although astrocytes may contribute to disease by propagating prions (74–76), only neurons degenerate in prion disease, and neurotoxicity is cell autonomous: neurons that do not express PrP are protected even if they are in direct contact with misfolded prions produced by neighboring cells (76–78). In contrast, neuroinflammatory responses from astrocytes and microglia (79–83) appear to be strictly non-autonomous, requiring neuronal prion infection (76).

That PrP-lowering ASOs extend survival in prion-infected mice (32,52,84) implies that they must lower PrP in neurons; nevertheless, we felt it prudent to further examine this link to determine whether there might ever exist circumstances in which a bulk tissue readout would indicate PrP lowering despite little or no target engagement in neurons. It is reassuring, then, that across a range of experimental parameters—dosing regimens, times post-dose, ASO chemistries and gapmer configurations, targets, species and brain regions—we never identified a circumstance in which bulk tissue would misinform about PrP RNA having been lowered in neurons.

Of course, given the somewhat differing activity of ASOs in distinct CNS cell types and the potential for drug concentration gradients across the brain, no single compartment readout, such as CSF PrP, can accurately report on every disease-relevant cell in this whole brain disease. Still, our findings provide one pillar of support for the expectation that lowered CSF PrP in an ASO trial is reasonably likely to predict clinical benefit in individuals at risk for prion disease.

### Concluding notes

Our study, together with recent data from the mouse lung (31), establishes the feasibility of using single nucleus transcriptomics to quantify ASO target engagement across cell types and individual cells within a bulk tissue. Our results answer key questions about ASO activity in the CNS, and support the utility of a CSF target engagement biomarker. Nonetheless, our studies were of limited scope relative to the number of questions that could be asked. Future studies of oligonucleotide drugs, particularly of new delivery routes, formulations, or conjugates, should consider single cell or single nucleus transcriptomics as tools for evaluating cell type specificity and single cell distribution.

## Supplementary Material

gkad371_Supplemental_Files

## Data Availability

A public git repository is available at https://github.com/ericminikel/scaso (permanent DOI: 10.5281/zenodo.7819353) containing all source code and a minimum analytical dataset (∼150 MB) sufficient to reproduce Figures 2–6, S1–S3, and all Supplementary tables and statistics in this manuscript. The full dataset (∼2 TB) is available at https://singlecell.broadinstitute.org/single_cell/study/SCP2126.
