# Supplementary material for: A single-cell map of antisense oligonucleotide activity in the brain
Source: Nucleic Acids Res. 2023 May 16;51(14):7109–24. doi: 10.1093/nar/gkad371 (PMC10415122; doi:10.1093/nar/gkad371)
Supplement: gkad371_Supplemental_Files [file gkad371_supplemental_files.zip › 2023-04-11-supplement.pdf]

SUPPLEMENTARY MATERIALS

A single-cell map of antisense oligonucleotide activity in the brain

Meredith A Mortberg[1*], Juliana E Gentile[1*], Naeem Nadaf[1], Charles Vanderburg[1], Sean Simmons[1], Dan Dubinsky[2], Adam Slamin[2], Salome Maldonado[2], Caroline L Petersen[2], Nichole Jones[2], Holly B Kordasiewicz[3], Hien T Zhao[3], Sonia M Vallabh[1,4,5,6†], Eric Vallabh Minikel[1,4,5,6†]

1. Stanley Center for Psychiatric Research, Broad Institute of MIT and Harvard, Cambridge, MA, 02142, USA
2. Genomics Platform, Broad Institute of MIT and Harvard, Cambridge, MA, 02141, USA
3. Ionis Pharmaceuticals, Carlsbad, CA, 92010, USA
4. McCance Center for Brain Health and Department of Neurology, Massachusetts General Hospital, Boston, MA, 02114, USA
5. Department of Neurology, Harvard Medical School, Boston, MA, 02115, USA
6. Prion Alliance, Cambridge, MA, 02139, USA

*equal contribution
†correspondence to: svallabh@broadinstitute.org or eminikel@broadinstitute.org

Supplementary Tables

Tables S1 - S23 are available in Excel format or as tab-separated text files at https://github.com/ericminikel/scaso (permanent DOI: 10.5281/zenodo.7819353).

Supplementary Figures

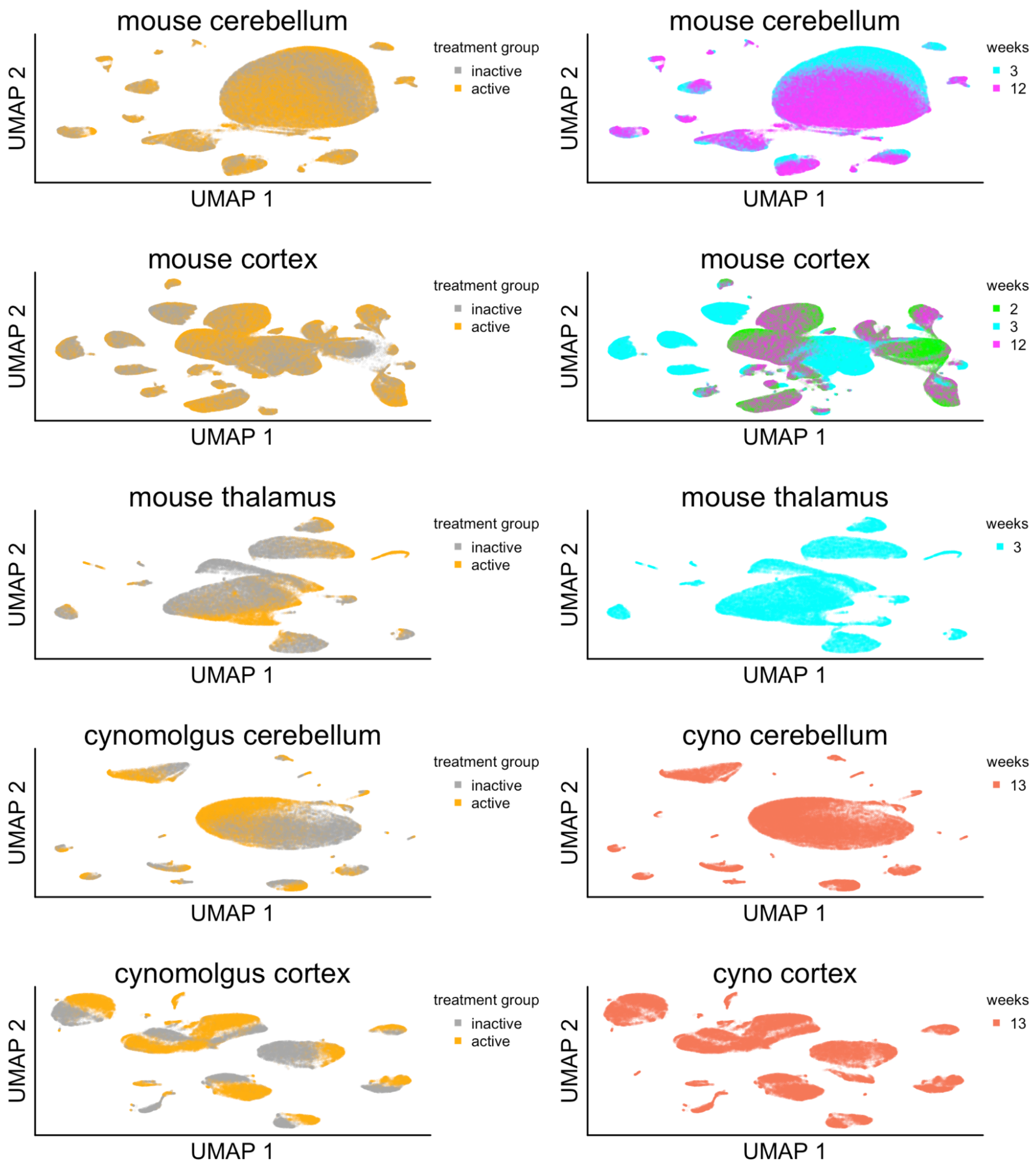

Figure S1. Weeks post-dose and treatment group in UMAP space. *UMAP plots from Figure 1 colored by treatment group (left) or weeks post-dose (right). Twinning of some cell types, particularly in mouse cortex, is due to a batch effect between 3-week versus 2- and 12-week post-dose animals; clusters are generally well-balanced between active and inactive treatment groups.*

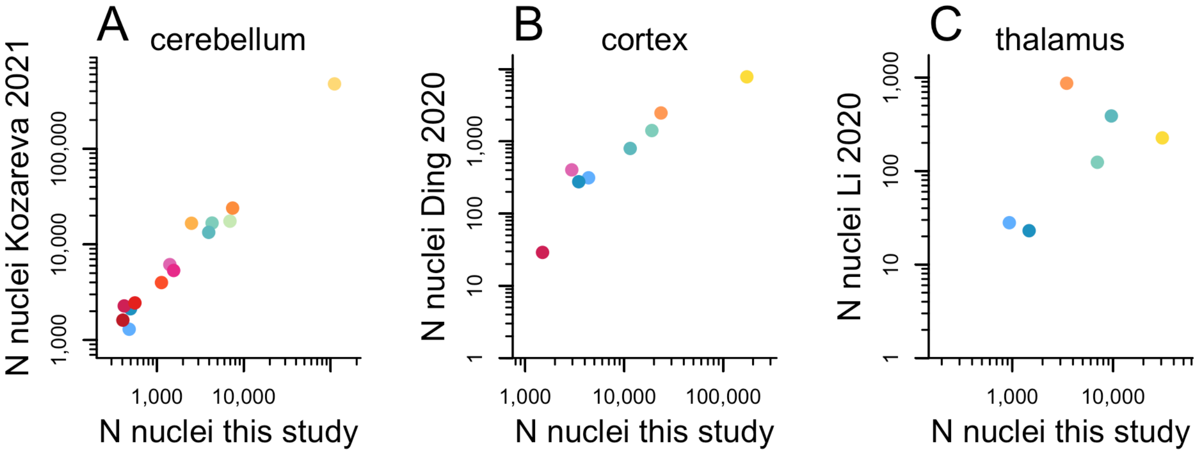

Figure S2. Correlation between number of nuclei of each cell type in our study versus previous work. *Number of cells in mouse brain regions in this study (x axis) versus reference datasets (y axis):* ***A)*** *cerebellum[30], rho > 0.99 ,P = 5.2e-19 (Pearson's correlation),* ***B)*** *cortex[48], rho = 0.98, P = 2.1e-6. Data for these plots are in Tables S3-S5.* ***C)*** *thalamus[38], rho = 0.20, P = 0.61 but note that reference dataset is thalamic reticular nucleus only, which is enriched for inhibitory neurons; excluding this cell type, rho = 0.63, P = 0.092,*

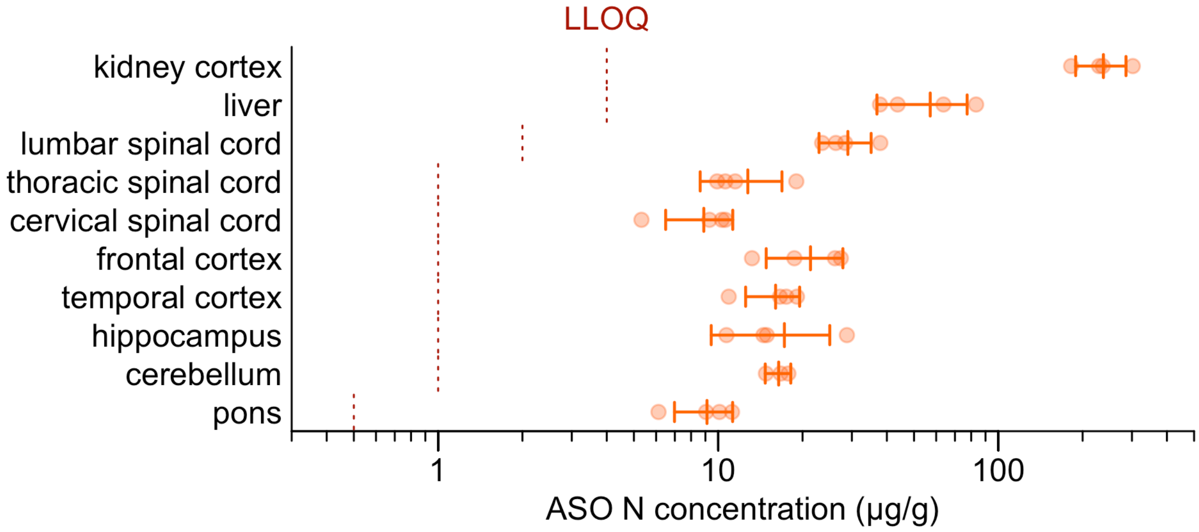

Figure S3. Pharmacokinetic analysis of NHP tissue. *Dots indicate individual animals (N=4 for each region except cerebellum, N=3), line segments indicate means and error bars indicate 95% confidence intervals of the mean. Different tissues were run at different dilutions, resulting in different lower limits of quantification (LLOQ), indicated by the dashed red lines. Note that frontal cortex is the region used in this study and referred to as "cortex" throughout Figure 5.*
